# Evaluation of the PETsys TOFPET2 ASIC in multi-channel coincidence experiments

**DOI:** 10.1186/s40658-021-00370-x

**Published:** 2021-03-24

**Authors:** Vanessa Nadig, David Schug, Bjoern Weissler, Volkmar Schulz

**Affiliations:** 1 Department of Physics of Molecular Imaging Systems, Experimental Molecular Imaging, RWTH Aachen University, Pauwelsstrasse 17, Aachen, 52074 Germany; 2 Hyperion Hybrid Imaging Systems GmbH, Pauwelsstrasse 19, Aachen, 52074 Germany; 3 III. Physikalisches Institut B, RWTH Aachen University, Otto-Blumenthal-Straße, Aachen, 52074 Germany; 4 Fraunhofer Institute for Digital Medicine MEVIS, Forckenbeckstrasse 55, Aachen, 52074 Germany

**Keywords:** Time-of-flight, Application-specific integrated circuits, ASIC, Positron emission tomography, PET, Coincidence resolution time, CRT, Energy resolution, TOFPET2

## Abstract

**Background:**

Aiming to measure the difference in arrival times of two coincident *γ*-photons with an accuracy in the order of 200ps, time-of-flight positron emission tomography systems commonly employ silicon photomultipliers (SiPMs) and high-resolution digitization electronics, application specific integrated circuits (ASICs). This work evaluates the performance of the TOFPET2 ASIC, released by PETsys Electronics S.A. in 2017, dependent on its configuration parameters in multi-channel coincidence measurements.

**Methods:**

SiPM arrays fabricated by different vendors (KETEK, SensL, Hamamatsu, Broadcom) were tested in combination with the ASIC. Scintillator arrays featuring different reflector designs and different configurations of the TOFPET2 ASIC software parameters were evaluated. The benchtop setup used is provided with the TOFPET2 ASIC evaluation kit by PETsys Electronics S.A.

**Results:**

Compared to existing studies featuring the TOFPET2 ASIC, multi-channel performance results dependent on a larger set of ASIC configuration parameters were obtained that have not been reported to this extend so far. The ASIC shows promising CRTs down to 219.9 ps in combination with two Hamamatsu S14161-3050-HS-08 SiPM arrays (128 channels read out, energy resolution 13.08%) and 216.1 ps in combination with two Broadcom AFBR-S4N44P643S SiPM arrays (32 channels read out, energy resolution 9.46%). The length of the trigger delay of the dark count suppression scheme has an impact on the ASIC performance and can be configured to further improve the coincidence resolution time. The integrator gain configuration has been investigated and allows an absolute improvement of the energy resolution by up to 1% at the cost of the linearity of the energy spectrum.

**Conclusion:**

Measuring up to the time-of-flight performance of state-of-the-art positron emission tomography (ToF-PET) systems while providing a uniform and stable readout for multiple channels at the same time, the TOFPET2 ASIC is treated as promising candidate for the integration in future ToF-PET systems.

## Background

A functional imaging modality widely used in the diagnosis and staging of cancer as well as in cardiology or neurology is positron emission tomography (PET) [1–3]. After an injected tracer undergoing a *β*^+^-decay, the emitted positron annihilates with an electron in the surrounding tissue, resulting in the back-to-back release of two *γ*-photons. The *γ*-photons are detected by two opposing elements of a ring-shaped detector, each consisting of a scintillator array coupled to a photo-detector array. Via a scintillation process, the *γ*-photons are stopped and converted in to optical photons, which then reach the employed photo-detector.

In time-of-flight positron emission tomography (ToF-PET), the difference in arrival times of the two detected *γ*-photons can be resolved, which results in a more precise localization of the annihilation event along the line of response (LOR) connecting the two points of detection [4, 5]. The coincidence resolution time (CRT) assesses the capability of a PET system to resolve this ToF information. Studies have shown that incorporating ToF information into the image reconstruction process increases the signal-to-noise ratio (SNR) of a PET image and therefore improves the image quality [6–8]. For a precise measurement of the ToF information, CRTs in the order of few hundred picoseconds are required. A low energy resolution in the order of 10% around 511 keV is of advantage to filter true coincidences from scattered events.

State-of-the-art clinical PET systems reach CRTs ranging from 214 ps to 500 ps. The achieved energy resolutions lay between 9% and 12% [5, 9, 10]. For prototype PET systems, CRTs between 200 ps to 450 ps and energy resolutions between 11 and 12% are reported [5, 11]. For both cases, future systems aim to reach CRTs below 200 ps [12–14]. On benchtop level, this limit has already been excelled by various setups reaching CRTs below 100 ps [15–17].

Scintillators used in PET systems require a high stopping power, a short scintillation decay time, and high photon statistics [18]. Reflective foils or mixtures of glues and powders can be used to optically segment an array of scintillators. Here, various scintillator topologies can be considered, namely one-to-one coupling between single scintillator needles and photo-sensor channels, multiple scintillator needles on one photo-sensor channel as well as monolithic scintillator blocks or slabs on an array of photo-sensor channels. The latter ones allow for depth-of-interaction (DOI) positioning [19–24].

In common state-of-the-art systems, photo-detectors such as photo-multiplier tubes (PMTs) and analog or digital silicon photomultipliers (SiPMs) and custom-designed readout electronics are employed. SiPMs became popular due to the high photo-detection efficiency (PDE) (up to 50%, some up to 65%), their high internal gain, their compactness, their fast response time, and their compatibility with magnetic fields, allowing simultaneous PET/MR imaging [17, 25–28]. SiPMs consist of several thousands single-photon avalanche diodes (SPADs) which are connected in parallel and operated in Geiger-mode. An incident optical photon hitting a SPAD causes a self-sustaining charge carrier avalanche. The avalanche effect is used for signal amplification enabling the detection of single optical photons, whereby individual SPAD signals are overlaid to a sum signal forming an SiPM pulse with a steep rising edge [25]. The first prototype system making use of digital SiPMs (dSiPMs) featuring direct binary digitization has been developed in recent years [29–33].

Typically, application-specific integrated circuits (ASICs) are employed to precisely digitize analog SiPM signals. This study specifically focuses on the application of ASICs in ToF-PET systems. Apart from exclusive ToF-PET systems, also hybrid imaging systems combining ToF-PET with other modalities, such as magnetic resonance imaging (MRI), electroencephalography (EEG) or ultrasound imaging (US), feature ASICs as readout and digitization electronics (MADPET4 [34, 35], TRIMAGE [36–38], EndoToFPET-US [13, 39]). ASICs typically feature a comparator with a low threshold to trigger on the rising edge of the SiPM pulse, i.e., on the first optical photons arriving, and a time-to-digital converter (TDC) to assign a timestamp to an event. The energy of the respective event can then be measured either by signal integration featuring capacitors (qdc-method) or by measuring the time over a specified threshold (tot-method) [13, 40, 41]. The Weeroc series [36, 37, 42], the PETA series [27, 43–48] and the TOFPET2 series by PETsys Electronics S.A. [49, 50] use such a qdc-method to measure the signal energy.

In this work, we evaluate the multi-channel performance of the TOFPET2 ASIC (version 2b), released by PETsys Electronics S.A. in 2017 [51–53]. Existing studies on the TOFPET2 ASIC performance deal with simulations or experimental results for single SiPMs in combination with the ASIC, which greatly differs to the experimental multi-channel results we are providing [12, 52, 54]. Further studies show multi-channel results that are restricted to a very small parameter space or focus on the DOI resolution of a certain scintillator topology obtained with TOFPET2 readout [53, 55–57]. They do not report experimental performance results dependent on the TOFPET2 configuration parameters, e.g., on the delay line configuration or the integrator gain, to the extend we do.

The goal of this study is to assess whether the TOFPET2 ASIC should be considered as solution for digitizing and processing analog SiPM signals in future (whole-body) ToF-PET systems. The presented multi-channel studies are necessary to investigate detector-related effects such as crosstalk and light-sharing as well as the channel-spread of the ASIC performance handling data from multiple channels at once, which cannot be assess in single-channel measurements. The compatibility of the ASIC with different SiPM types in combination with different materials for scintillator segmentation was tested. In addition, the parameters of the ASIC configuration related to the trigger circuit were varied. The impact of the hard- and software configuration on the ASIC performance was quantified.

## Materials and methods

### TOFPET2 ASIC

The TOFPET2 ASIC is characterized by its compactness (14 mm × 14 mm chip size including bonding area [58]), 128 readout channels, low power consumption [49, 59] and a high hit rate of up to 480 kcps per channel, which corresponds to a data rate of 640 Mbit/s [49, 54]. The maximum output data rate of the ASIC, i.e., 64 channels, is specified as 2.8 Gbit/s [49].

Each of the 64 individual channels is multi-buffered by four analog buffers and employs a three-threshold trigger logic with two discriminators D_T1 and D_T2 in the timing and one discriminator D_E in the energy branch [49, 60, 61]. A global- and a channel-specific configuration register allow to change the ASIC configuration. The ASIC can be operated in a time-over-threshold (tot) or an energy integration (qdc) mode for energy measurement. The charge-to-digital converter (QDC) used in qdc mode behaves linear for integration charges up to 1500 pC [54]. The TDC has a resolution of 30 ps. The chip runs with a clock cycle of 200 MHz.

**Fig. 1:**
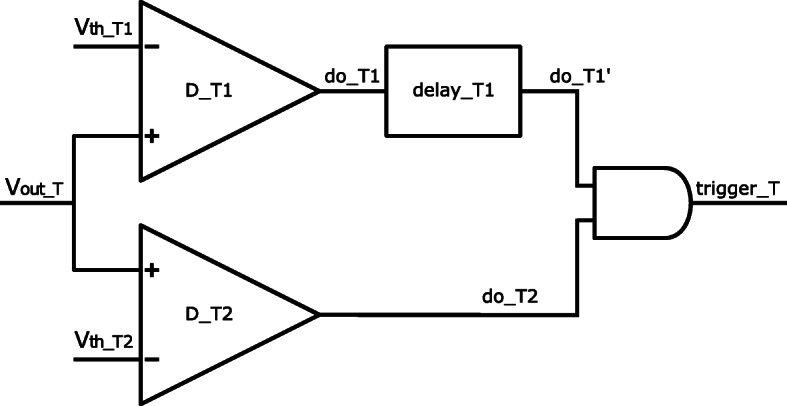
Schematic drawing of the TOFPET2 channel trigger circuit. In the timing branch of the trigger circuit, the output of the first discriminator D_T1 is delay by a configurable delay element with respect to the output of the second discriminator D_T2. The design allows the rejection of small noise pulses

Incoming SiPM signals are amplified by a trans-impedance ampliflier in the timing branch (nominal gain 3000 *Ω*) and a transimpedance ampliflier energy branch (nominal gain 300 *Ω*), respectively [49]. As determined in an oscilloscope measurement, the pre-amplified SiPM pulses in the timing branch have a signal height of about 300 mV. The thresholds of the three discriminators can be adjusted via the three dimensionless parameters *vth*_t1, *vth*_t2, and *vth*_e in the ASIC configuration. Increasing these parameters by one digital-to-analog converter (DAC) step is equal to increasing the trigger level by approximately 2.5 mV, 15 mV, and 20 mV, respectively, over a baseline set during calibration [62]. For a proper operation of the trigger logic [52], it has to be ensured that the voltage thresholds at the discriminators *Vth*_T1 and *Vth*_T2 fulfill *Vth*_T1<*Vth*_T2. The trigger circuit of each channel enables dark count rejection and high timing resolution by triggering on a low voltage threshold with the first discriminator D_T1 at a very early point in time, i.e., on the first optical photons hitting the SiPM. This trigger is delayed by a specified delay period and passes an AND gate opened by a second trigger activated on a higher voltage threshold with a second discriminator D_T2 (see Fig. 1). The delay period is in the order of few nanoseconds and can be configured by adjusting the parameter *fe*_*delay* of the ASIC configuration [49]. The design of the trigger logic allows the rejection of small noise pulses, but is associated with the generation of satellite peaks in the coincidence time difference spectra which are caused by a shift in timestamp generation from the delayed D_T1 to the non-delayed D_T2. A detailed description on the operation of the trigger circuit and the generation of satellite peaks is given in [52].

### Benchtop setup

To evaluate the TOFPET2 ASIC in combination with different SiPM types, we used the TOFPET2 ASIC evaluation kit designed by PETsys Electronics S.A. as it enables the user to test various ASIC-SiPM combinations under benchtop conditions. The kit comes along with two ASIC test boards each allowing to connect an SiPM array to a TOFPET2 ASIC via two SAMTEC connectors. A high-voltage digital-to-analog converter (HV-DAC) mezzanine board (version 08/2016) providing the bias voltage for the employed SiPMs, and a front end board (FEB/D) generating the global clock signal and holding the main power supply for the ASIC test boards are included as well. Data transmission to a readout computer is conducted via a 1-Gigabit Ethernet (GbE) mezzanine board. The ASIC test boards provide direct coupling between the employed SiPMs and the ASIC.

**Fig. 2:**
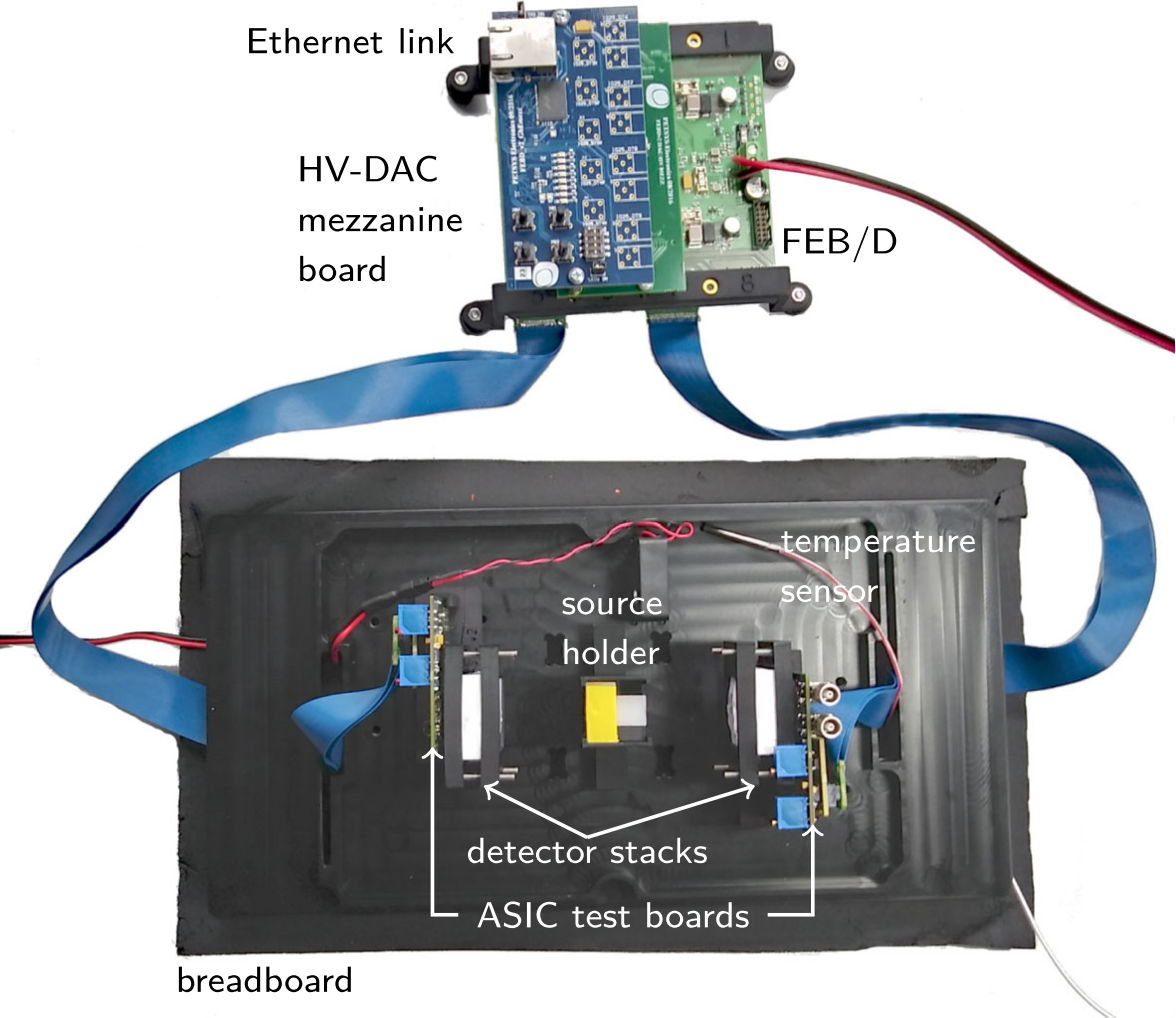
Benchtop setup. Optical breadboard holding two ASIC test boards equipped with KETEK PA3325-WB-0808 SiPMs for coincidence experiments. The whole setup can be enclosed with a top cover featuring thermo-control and operated inside a climate chamber. Graphic reprinted from [59]

**Fig. 3:**
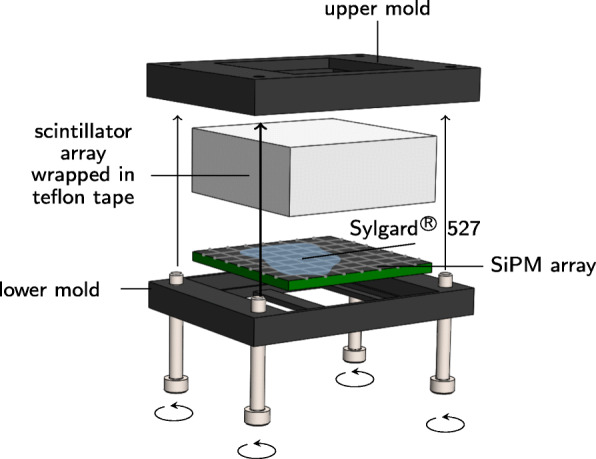
Assembly tools. Molds used to assemble the detector stacks composed of a segmented scintillator array, a coupling material, and an SiPM array. Each stack was wrapped in teflon tape to prevent light-loss

Additionally, a breadboard for mounting the setup is provided with the kit (see Fig. 2). To conduct coincidence experiments, the breadboard allows to position both ASIC test boards face-to-face in different distances to the source holder mounting position. Featuring two cable inlets, which were used to connect the ASIC test boards to the FEB/D board via two flexible cables, the breadboard can be encapsulated light-proof by a top cover. A temperature sensor and a proportional–integral–derivative (PID) controller regulating a thermo-element were used to adjust the temperature insight the box. In addition, the whole benchtop setup was placed in a climate chamber to ensure a stable temperature control. A custom-designed source holder was employed to assemble the multi-source geometry. Molds were used to assemble the crystal-SiPM array configurations (see Fig. 3).

### Detectors

To test the compatibility and performance of the TOFPET2 ASIC with different analog SiPM types, we used 8 ×8 SiPM arrays fabricated by different vendors. Two samples each of KETEK PA3325-WB-0808, SensL ArrayJ-30020-64P-PCB, Hamamatsu S14161-3050-HS-08, and Broadcom AFBR-S4N44P643S (see Table 1) were used to perform coincidence experiments. A multi-source geometry of five ^22^Na NEMA cube point sources with a total activity of approximately 3 MBq was placed in the center of the setup during these experiments. For the SensL ArrayJ-30020-64P-PCB SiPM arrays, additional adapter boards needed to be employed to establish the connection to the ASIC test board.

**Table 1:** Characteristics of the investigated analog SiPM types. Parameters are taken from the respective data sheet

	KETEK	SensL	Hamamatsu	Broadcom
	PA3325-WB-0808	Array-J-30020-64P-PCB	S14161-3050-HS-08	AFBR-S4N44P643S
Pitch	3.36 mm	3.36 mm	3.20 mm	3.93 mm
Active area	3.0 × 3.0 mm^2^	3.07 × 3.07 mm^2^	3.0 × 3.0 mm^2^	3.72 × 3.72 mm^2^
No. of SPADs	13920	14850	3531	15060
SPAD size	25 µm	20 µm	50 µm (pitch)	30 µm (pitch)
Breakdown voltage	27 V	24.2 V to 24.7 V	37 V	26.9 V
Gain	1.74·10^6^ (5 V)	1.9·10^6^ (5 V)	2.5·10^6^ (2.7 V)	3.3·10^6^ (7 V)
Dark count rate	100 kHz mm^2^ (5 V)	125 kHz mm^2^ (5 V)	-	270 kHz mm^2^ (7 V)
PDE	43% (5 V)	38% (5 V)	50% (2.7 V)	55% (7 V)
Reference	[78]	[79]	[80]	[81]

Using Sylgard® 527, a two-component dielectric gel fabricated by Dow Corning for optical coupling, each SiPM array was successively one-to-one-coupled to 8 × 8 or 4 × 4 LYSO scintillator arrays. These arrays feature different segmentation layers (360 µm or 110 µm BaSO_4_ powder mixed with epoxy and 67 µm glued enhanced specular reflector (ESR) foil. The size of an individual scintillator needle in the 8 × 8 ESR array is 3.28 mm × 3.28 mm × 12.00 mm. The size of an individual scintillator needle in the 4 × 4 ESR array is 3.85 mm × 3.85 mm × 12.00 mm. For thicker reflector layers, the scintillator width is reduced by the additional width of the reflector. The selected scintillator length was chosen to reduce the influence of time jitter related light-transport effects accompanying long scintillator needles [63], and thus, be more sensitive to ASIC-related effects on the timing performance. The chosen scintillator length can be found in some pre-clinical and clinical PET systems [5, 33]. A performance degradation is expected if operating the TOFPET2 ASIC in combination with longer scintillator needles. To give a benchmark of this degradation, additional measurements were conducted using a 4 × 4 ESR array consisting of 2.62 mm × 2.62 mm × 19.00 mm scintillator needles.

### Setup calibration

The setup was calibrated using the PETsys calibration routine implemented in the software coming along with the evaluation kit [61]. For each SiPM type, a calibration was run once at default ASIC configuration applying an overvoltage of 4 V at an environment temperature of 16^∘^C. The required bias voltages were specified depending on the SiPM type employed. It is not necessary to calibrate the setup for every threshold or overvoltage change. A radioactive source can be left in the setup during calibration since this does not affect the determined parameters [62].

### Parameter studies

For each SiPM-crystal configuration, the discriminator threshold *vth*_t1 was varied between 10 and 50 in steps of 10, while *vth*_t2 and *vth*_e were kept at constant values (*vth*_t2=20, *vth*_e=15). This roughly corresponds to triggering between the 1 p.e. to 3 p.e. (photoelectron) as it is reported for the example of a Hamamatsu S14161-3050-HS-08 SiPM array in [59]. For each setting, raw data were acquired for 120 s at an overvoltage of 2.75 V to 7.75 V. Additionally, the crystal top-to-source distance was varied between 18 mm to 58 mm in steps of 20 mm.

For the KETEK PA3325-WB-0808 arrays coupled to the scintillator arrays featuring 360 µm BaSO_4_ as segmentation layer, the trigger delay period was configured as different lengths in the range of few nanoseconds (0.0 ns to 12.9 ns) for *vth*_t1=10 and *vth*_t1=50 and overvoltages between 2.75 V to 7.75 V. For the Hamamatsu S14161-3050-HS-08 arrays coupled to the scintillator arrays featuring 360 µm BaSO_4_ as segmentation layer, the integrator gain settings *G*_*Q*1_ and *G*_*Q*2_ were varied between 0.32-2.25 and 1.00-1.68, respectively, to evaluate the linearity of the acquired energy value spectra and the influence on the resulting energy resolution. For each setting, raw data were acquired for 120 s at an overvoltage of 4.75 V and with *vth*_t1=20, *vth*_t2=20, and *vth*_e=15. Effects due to the assembly of SiPM and scintillator array can be excluded since groups of measurements varying the trigger delay and gain configuration as well as the discriminator thresholds are performed with the same assembly directly after each other.

### Data collection and processing

For data collection, we used the data acquisition routine implemented by PETsys. We prepared the acquired raw data with the *convert*_*raw*_*to*_*singles* method implemented by PETsys and provided with the evaluation kit [61]. Using this routine, raw data were converted into single-raw-hit information by applying the acquired calibration data. A table containing a timestamp, an energy value, and a channel id for each single event acquired was returned. The returned energy value does not correspond to ADC bins on hardware level, but is given in an arbitrary unit.

These single events were further processed using an analysis software developed at our institute [52]. The energy values returned by the PETsys routine were sorted into channel-individual energy value histograms. Afterwards, the noise background of this spectrum was estimated and subtracted [64–66]. The positions of the 511 keV and 1274.5 keV peaks were determined applying a Gaussian peak finder routine and an iterative Gaussian fitting method after subtracting the background of the spectrum [67]. A simple saturation model neglecting offsets and optical crosstalk was applied to the found positions, parametrizing the saturation corrected energy as

1 $$ E = c \cdot s \cdot \log \left(\frac{1}{1 - \frac{e}{s}} \right)  $$

where *E* is the energy in keV, *c* is a correction factor in calibrated energy units ^−1^, *s* is a saturation factor in calibrated energy units, and *e* is the acquired energy value in calibrated energy units [52], similar to [12]. Now, all acquired energy values are converted and sorted into channel-individual energy histograms. An energy filter ranging from 400 keV to 700 keV was applied to filter true coincidences, which should have an energy around 511 keV, from scattered events. A Gaussian was fitted to the remaining histogram data corresponding to the 511 keV peak. The energy resolution was determined as the full width at half maximum (FWHM) of the fitted Gaussian summing up all channel-individual energy histograms to a global energy histogram. The fit range was iteratively adjusted by 10% of the FWHM. Filtered events were checked for coincidences applying a coincidence window of 7.5 ns. To evaluate the effects of different trigger delay configurations, this window was extended to 35 ns. The time difference between two events matched as a coincidence was calculated. All computed time differences were filled into a time difference histogram. A Gaussian was fitted to the histogram peak while iteratively adjusting the fit range by 10% of the FWHM. The coincidence resolution time (CRT) was determined as the FWHM of the fitted Gaussian. Additionally, the full width at tenth maximum (FWTM) is computed to get information about the tail behavior of the peak. A Gaussian excess factor *gaussexc* is used to specify the outgrow of the tails with reference to their expected behavior. This factor is defined as [24]

2 $$ \mathsf{gaussexc} = \frac{\mathsf{FWTM} - \frac{\mathsf{FWHM}}{2.355} \cdot 4.294}{\mathsf{FWTM}}  $$

where *FWHM*/2.355·4.294 computes the theoretical value of the FWTM for a truly Gaussian distribution. The obtained performance parameters were always plotted as a function of the offset-corrected overvoltage $U_{\mathsf {ov\_cor}}$ computed via

3 $$ U_{\mathsf{ov\_cor}} = U_{\mathsf{ov,set}} - U_{\mathsf{off}}  $$

The overvoltage voltage set *U*_*ov*,*set*_ via the acquisition routine was corrected by an offset *U*_*off*_ (approximately 750 mV [68]).

## Results

**Fig. 4:**
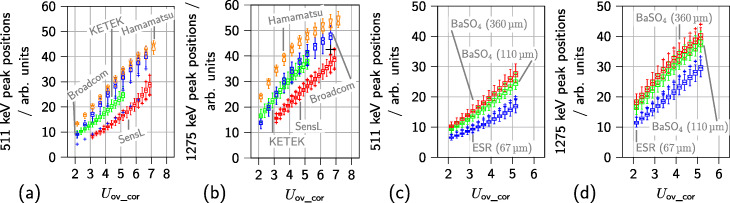
Spread in gain for 128 ASIC channels coupled to SiPM arrays. A geometry of five NEMA cubes (^22^Na sources) with a total activity of approximately 3 MBq is employed. **a** Position of the 511-keV peak in the energy value spectrum for different SiPM types. **b** Position of the 1275-keV peak in the energy value spectrum. Measurements were conducted using 110 µm BaSO_4_ as segmentation layer and SiPM arrays from Broadcom, KETEK, SensL, and Hamamatsu. **c** Position of the 511-keV peak in the energy value spectrum for different segmentation layers. **d** Position of the 1275-keV peak in the energy value spectrum for different segmentation layers. Measurements were conducted with two KETEK PA3325-WB-0808 SiPM arrays. All positions are plotted against the applied offset-corrected overvoltage $\mathsf {U_{ov\_cor}}$

**Fig. 5:**
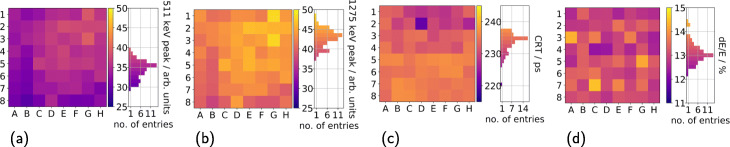
Uniform channel performance. A geometry of five NEMA cubes (^22^Na sources) with a total activity of approximately 3 MBq is employed. **a** Position of the 511-keV peak in the energy value spectrum. **b** Position of the 1275-keV peak in the energy value. **c** Coincidence resolution time (CRT) computed as the mean of the CRTs of this channel with all its coincident channels. **d** Energy resolution (dE/E). Measurements were conducted with two Hamamatsu S14161-350-HS-08 SiPM arrays at 4 V overvoltage and *vth*_t1=50. BaSO_4_ powder (360 µm) mixed with an epoxy was used as segmentation layer. The scintillator array used consists of 8 × 8 needles with a size of 3 mm × 3 mm × 12 mm

The energy value spectra of different SiPM types allow to determine 511-keV and 1275-keV peak positions for all operated channels. The peaks are shifted to higher energy values for higher overvoltages (see Fig. 4a, b). The spread of the positions observed for individual channels increases for higher overvoltages and the peak positions are sorted according to the SiPM gains (see Table 1). In addition, the peaks are shifted to higher energy values if a thicker segmentation layer is used in the scintillator array and if BaSO_4_ is used instead of ESR (see Fig. 4c, d). The spatial structure of the channel spread of the peak positions as well as the determined energy resolution and CRT is shown exemplary for a Hamamatsu S14161-3050-HS-08 array in Fig. 5. The channel spread in peak position and energy resolution shows a Gaussian behavior with few outliers. For the CRTs, which were calculated as mean of the CRTs of the respective channel with its coincident channels, a longer tail of the distribution is visible towards lower values.

**Fig. 6:**
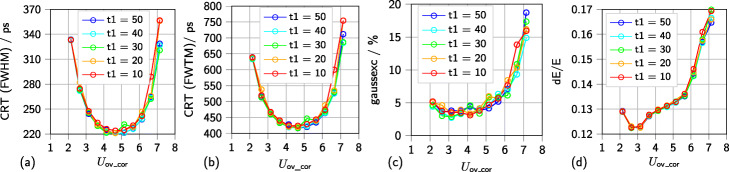
Impact of the trigger threshold on CRT and energy resolution. Measurements were conducted with two Hamamatsu S14161-3050-HS-08 each coupled to an 8 × 8 12-mm-high LYSO scintillator array featuring 360 µm BaSO_4_ powder mixed with epoxy as segmentation layer. Single scintillator needles had a size of 3 mm × 3 mm × 12 mm. A geometry of five NEMA cubes (^22^Na sources) with a total activity of approximately 3 MBq is employed. **a** Coincidence resolution time (CRT - FWHM). **b** Coincidence resolution time (CRT - FWTM). **c** Gaussian excess factor (*gaussex*). **d** Energy resolution (dE/E)

Determining these performance parameters globally, operating two Hamamatsu S14161-3050-HS-08 SiPM arrays at a range of overvoltages shows an improvement in CRT (FWHM and FWTM) and energy resolution for higher overvoltages before deteriorating again (see Fig. 6). The reported Gaussian excess factor *gaussexc* show an increased contribution of non-Gaussian tails to the time difference spectra for higher overvoltages (see Fig. 6c). Triggering on higher thresholds *vth*_t1 further improves the CRT (FWHM and FWTM). The energy resolution is rarely affected by this parameter change. Generally, these effects are observed for all investigated SiPM types and segmentation layers. The point of transition from the negative to the positive influence of higher overvoltages and discriminator thresholds *vth*_t1 varies according to the SiPM type and scintillator array used.

**Fig. 7:**
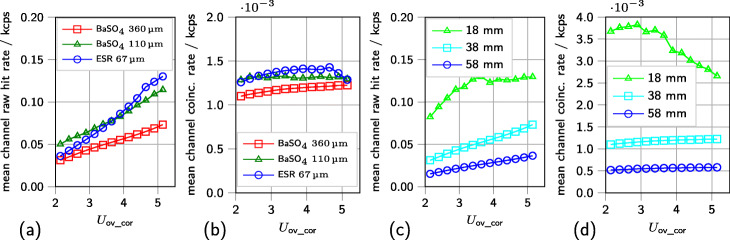
Data rates for different setup hardware configurations. **a** Mean raw hit rate per channel for different reflector materials. **b** Mean coincidence rate per channel for different reflector materials. **c** Mean raw hit rate per channel for different distances of the detectors to the source. **d** Mean coincidence rate per channel for different distances of the detectors to the source

To compare the influence of the investigated parameters, the lowest CRT (FWHM) achieved with each configuration along the investigated range of overvoltages is reported together with the corresponding energy resolution in Tables 2 and 4. Depending on the SiPM type, changing the threshold *vth*_t1 results in a CRT (FWHM) improvement of approximately 3 ps to 9 ps (approximately 1% to 3% relative improvement). Triggering on a higher threshold, also leads to slightly lower Gaussian excess factors, i.e., a more Gaussian behavior of the tails of the coincidence time difference histogram, especially for KETEK and SensL SiPMs (see Table 3). Among the different scintillator arrays, both scintillator types employing BaSO_4_ powder mixed with epoxy as segmentation layer outperform the type featuring ESR foil. A segmentation layer of 360 µm BaSO_4_ reaches approximately 100 ps to 200 ps lower CRTs (FWHM) than a segmentation layer of 67 µm ESR foil (see Table 2). This is equal to a relative performance gain of approximately 29% to 43% depending on the SiPM type. The CRT (FWTM) shows a similar behavior (see Table 3). Comparing the corresponding energy resolutions, an absolute improvement of up to approximately 2.5% can be reported (see Table 4). Here, the thicker BaSO_4_-layer of 360 µm shows better performance results (approximately 6% to 14% relative performance gain) than the thinner layer of 110 µm. For different segmentation layers, different mean channel raw hit rates and mean channel coincidence rates, respectively, are reported (see Fig. 7a, b).

**Table 2:** Coincidence resolution time (FWHM)

	KETEK	SensL	Hamamatsu
	PA3325-WB-0808	Array-J-30020-64P-PCB	S14161-3050-HS-08
Threshold *vth*_t1			
10	273.8±0.7 ps	265.2±0.6 ps	224.1±0.7 ps
20	271.4±0.7 ps	265.4±0.6 ps	221.2±0.7 ps
30	268.6±0.7 ps	263.7±0.6 ps	221.1±0.7 ps
40	266.1±0.6 ps	262.8±0.6 ps	219.9±0.7 ps
50	264.6±0.6 ps	262.4±0.6 ps	220.0±0.7 ps
Segmentation layer			
BaSO_4_ (360 µm)	264.6±0.6 ps	262.4±0.6 ps	220.0±0.7 ps
BaSO_4_ (110 µm)	282.8±0.6 ps	306.5±0.6 ps	242.9±0.5 ps
ESR (67 µm, glued)	371.4±0.8 ps	463.4±0.8 ps	328.1±0.7 ps
Distance			
18 mm	277.3±0.4 ps	-	-
38 mm	264.6±0.6 ps	262.4±0.6 ps	220.0±0.7 ps
58 mm	245.3±0.8 ps	253.3±0.8 ps	224.4±0.7 ps
Clinical (19 mm height)			
ESR (155 µm, air-coupled)	317.3±1.9 ps	325.1±1.7 ps	247.5±2.4 ps

Parameter study for multi-channel configurations featuring SiPMs by different vendors. Table reports the lowest CRT achieved in coincidence measurements of two times 64 channels with the corresponding settings and materials used. If not indicated differently, data were acquired at *vth*_t1=50, *vth*_t2=20, *vth*_e=15, with 360 µm BaSO_4_ for scintillator segmentation, a scintillator height of 12 mm, and at a distance of 38 mm. The overvoltage setting varies according to the parameter setting and SiPM type used. For the clinical configuration (19-mm high scintillator), only two times 16 channels were read out

**Table 3:** Coincidence resolution time (FWTM) and Gaussian excess factors *gaussexc*

	KETEK		SensL		Hamamatsu	
	PA3325-WB-0808		Array-J-30020-64P-PCB		S14161-3050-HS-08	
	CRT (FWTM)	gaussexc	CRT (FWTM)	gaussexc	CRT (FWTM)	gaussexc
Threshold *vth*_t1						
10	524.6 ps	5.07%	528.9 ps	9.38%	424.1 ps	3.80%
20	514.2 ps	3.89%	514.6 ps	6.34%	420.1 ps	4.13%
30	511.4 ps	4.40%	517.2 ps	7.56%	416.7 ps	3.36%
40	501.6 ps	3.40%	504.3 ps	5.22%	417.0 ps	4.02%
50	503.6 ps	4.39%	507.7 ps	6.12%	416.4 ps	3.82%
Segmentation layer						
BaSO_4_ (360 µm)	503.6 ps	4.39%	507.7 ps	6.12%	416.4 ps	3.82%
BaSO_4_ (110 µm)	542.6 ps	5.23%	604.9 ps	5.29%	464.9 ps	4.97%
ESR (67 µm, glued)	715.1 ps	5.61%	896.7 ps	6.13%	609.6 ps	8.37%
Distance						
18 mm	539.6 ps	6.71%	-	-	-	-
38 mm	503.6 ps	4.39%	507.7 ps	6.12%	416.4 ps	3.82%
58 mm	463.8 ps	3.68%	477.2 ps	3.32%	419.9 ps	2.64%
Clinical (19 mm height)						
ESR (155 µm, air-coupled)	601.9 ps	4.04%	617.2 ps	4.11%	462.3 ps	2.43%

Parameter study for multi-channel configurations featuring SiPMs by different vendors. Table reports the CRT (FWTM) and Gaussian excess factors corresponding to the lowest CRTs achieved in coincidence measurements of two times 64 channels (see Table 2). If not indicated differently, data were acquired at *vth*_t1=50, *vth*_t2=20, *vth*_e=15, with 360 µm BaSO_4_ for scintillator segmentation, a scintillator height of 12 mm, and at a distance of 38 mm. The overvoltage setting varies according to the parameter setting and SiPM type used. The error on the reported CRTs (FWTM) is less than 0.1 ps. For the clinical configuration (19-mm high scintillator), only two times 16 channels were read out

**Table 4:** Energy resolution

	KETEK	SensL	Hamamatsu
	PA3325-WB-0808	Array-J-30020-64P-PCB	S14161-3050-HS-08
Threshold *vth*_t1			
10	10.35±0.02%	11.20±0.02%	13.10±0.02%
20	10.44±0.02%	10.92±0.02%	13.15±0.03%
30	10.84±0.02%	11.22±0.02%	13.12±0.03%
40	10.85±0.02%	11.06±0.02%	13.08±0.03%
50	11.08±0.02%	11.23±0.02%	13.13±0.03%
Segmentation layer			
BaSO_4_ (360 µm)	11.08±0.02%	11.23±0.02%	13.13±0.03%
BaSO_4_ (110 µm)	12.60±0.02%	11.64±0.02%	14.70±0.02%
ESR (67 µm, glued)	12.58±0.02%	13.78±0.02%	14.74±0.02%
Distance			
18 mm	10.94±0.01%	-	-
38 mm	11.08±0.02%	11.23±0.02%	13.13±0.03%
58 mm	10.67±0.02%	10.66±0.02%	11.39±0.03%
Clinical (19 mm height)			
ESR (155 µm, air-coupled)	10.74±0.05%	10.72±0.04%	10.46±0.08%

Parameter study for multi-channel configurations featuring SiPMs by different vendors. Table reports the energy resolution corresponding to the lowest CRTs achieved in coincidence measurements of two times 64 channels (see Table 2). If not indicated differently, data were acquired at *vth*_t1=50, *vth*_t2=20, *vth*_e=15, with 360 µm BaSO_4_ for scintillator segmentation, a scintillator height of 12 mm, and at a distance of 38 mm. The overvoltage setting varies according to the parameter setting and SiPM type used. For the clinical configuration (19-mm high scintillator), only two times 16 channels were read out

Triggering at larger distances between sensor and the employed multi-source geometry leads to an improvement of the CRT (FWHM) and energy resolution by about 10 ps to 20 ps and 1.7% for a distance variation of 20 mm (see Tables 2 and 4). Moving the detector stacks from the farthest to the closest distance corresponds to a three- to fourfold increase of the mean channel raw hit rate and an about seven-fold increase of the mean channel coincidence rate, measured at 2 V (see Fig. 7c, d).

Due to limited availability of materials, the parameter study was only partly performed for the Broadcom AFBR-S4N44P643S SiPM arrays. Here, triggering on a higher threshold *vth*_t1 improved the lowest CRT (FWHM) achieved by up to approximately 25 ps, which corresponds to approximately 9% relative performance gain (see Table 5). The corresponding CRT (FWTM) is improved as well. The corresponding energy resolution only showed minor fluctuations less than 0.3% (absolute change). Again, the scintillator type employing BaSO_4_ powder mixed with epoxy as segmentation layer outperform the type featuring ESR foil regarding the CRT.

**Table 5:** Supplementary results contributing to the parameter study

	Broadcom AFBR-S4N44P643S			
	CRT (FWHM)	CRT (FWHM)	gaussexc	dE/E
Threshold *vth*_t1				
10	240.2±2.9 ps	449.7 ps	2.68%	9.37±0.08%
20	232.6±2.8 ps	437.4 ps	3.15%	9.38±0.08%
30	223.5±2.7 ps	426.9 ps	4.78%	9.65±0.09%
40	220.1±2.6 ps	419.1 ps	4.45%	9.40±0.08%
50	216.1±2.6 ps	413.3 ps	4.92%	9.46±0.09%
Segmentation layer				
BaSO_4_ (110 µm)	216.1±2.6 ps	582.0 ps	4.23%	9.46±0.09%
ESR (67 µm, glued)	293.6±2.6 ps	413.3 ps	4.92%	11.64±0.08%

Table reports the lowest CRT achieved with the corresponding settings and materials used and the CRT (FWTM), Gaussian excess factors and energy resolution at the selected operation point. Data were acquired with two Broadcom AFBR-S4N44P643S SiPM array, where only 16 channels per array were read out. If not indicated differently, data were acquired at *vth*_t1=50, *vth*_t2=20, *vth*_e=15, with 110 µm BaSO_4_ for scintillator segmentation, a scintillator height of 12 mm, and at a distance of 58 mm. The overvoltage setting varies according to the parameter setting used

Among the four SiPM types tested in combination with the TOFPET2 ASIC, the 8 × 8 Hamamatsu S14161-3050-HS-08 SiPM arrays outperform the other SiPM arrays achieving CRTs (FWHM) down to 219.9 ± 0.7 ps (dE/E = 13.08 ± 0.03%) with an 8 × 8 12-mm-high scintillator array featuring 360 µm BaSO_4_ mixed with epoxy as segmentation layer for 128 channels read out. Broadcom AFBR-S4N44P643S SiPM arrays reached comparable CRTs down to 216.1 ± 2.6 ps (dE/E = 9.46 ± 0.09%) with a 4 × 4 12-mm-high scintillator array featuring a thinner BaSO_4_ layer (110 µm) for 32 channels read out. With a 4 × 4 19-mm-high scintillator array featuring 155 µm air-coupled ESR-foil as the inter-crystal layer, the Hamamatsu arrays reached CRTs (FWHM) down to 247.5 ± 2.4 ps (dE/E = 10.46 ± 0.08%) for 32 channels read out.

**Fig. 8:**
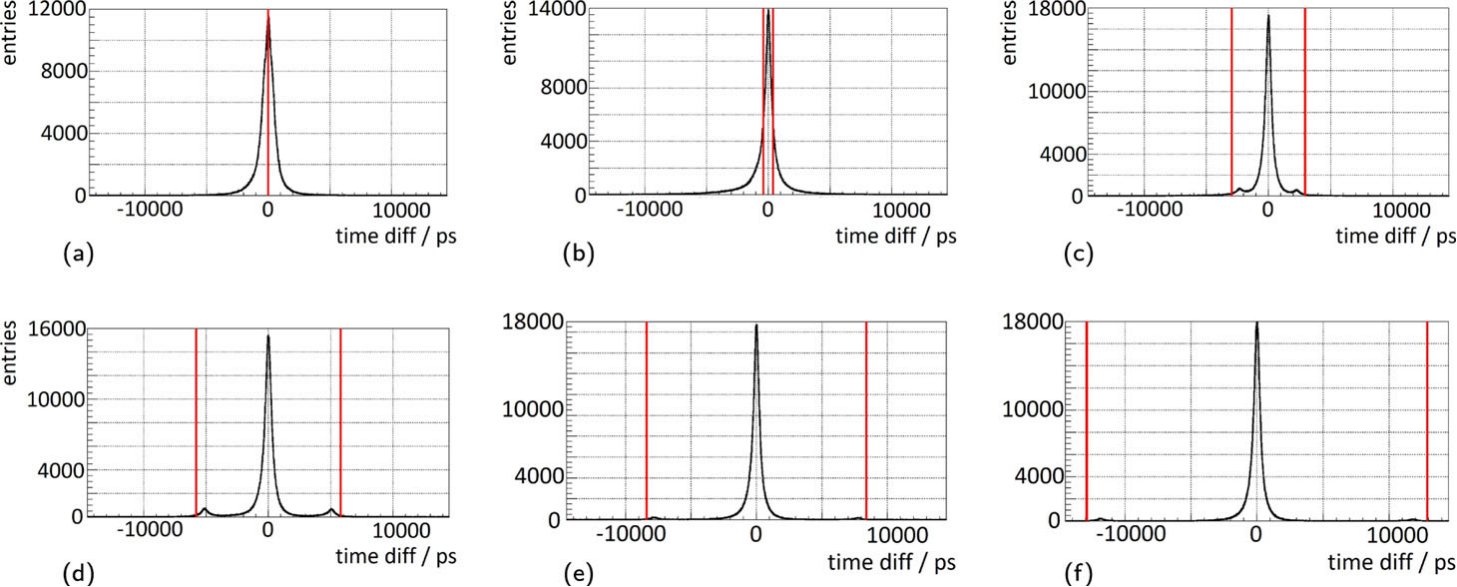
Satellite peaks in the coincidence time difference spectra for different delay periods. Measurements were conducted with two KETEK PA3325-WB-0808 each coupled to an 8 × 8 12-mm-high LYSO scintillator array featuring 360 µm BaSO_4_ powder mixed with epoxy as segmentation layer. Data are collected at 4.75 V overvoltage and with *vth*_t1=10. A geometry of five NEMA cubes (^22^Na sources) with a total activity of approximately 3 MBq is employed. Red lines indicate the configured delay period [49, 62]. **a** Delay line bypassed. **b** 0.39 ns. **c** 2.95 ns. **d** 5.8 ns. **e** 8.4 ns. **f** 12.9 ns

**Fig. 9:**
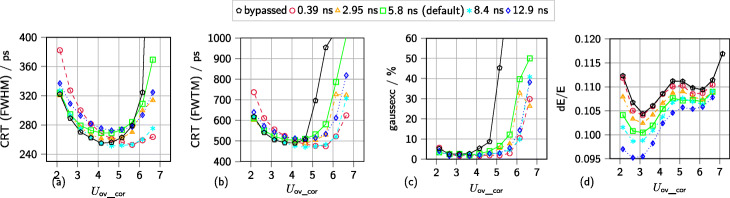
Performance results dependent on the configured delay period. Data are acquired with two KETEK PA3325-WB-0808 in multi-channel coincidence experiments with *vth*_t1=50. Each SiPM array is coupled to an 8 × 8 LYSO of 12 mm height scintillator array featuring 360 µm BaSO_4_ powder mixed with epoxy as the inter-crystal layer. A geometry of five NEMA cubes (^22^Na sources) with a total activity of approximately 3 MBq is employed. **a** Coincidence resolution time (CRT - FWHM). **b** Coincidence resolution time (CRT - FWTM). **c** Gaussian excess factor (*gaussex*). **d** Energy resolution (dE/E)

Satellite peaks, which were observed for measurements with single SiPMs [52], also appear in the coincidence time difference spectra of multi-channel measurements (see Fig. 8). The peaks are generated by small noise pulses that trigger the first discriminator and are validated by a true coincidence event triggering on the second discriminator occurring shortly after the noise event and while the *AND* gate connecting first and second discriminator is still active. As Fig. 8 depicts, the shift of the peaks from the center peak in the time difference histogram is related to the configured trigger delay between the first and second discriminator of the ASIC channel circuit. The fraction of events causing the formation of satellite peaks is higher for higher overvoltages and lower discriminator thresholds *vth*_t1. A broader explanation is given in [52]. As Fig. 9 depicts, changing the delay period between the first and second discriminator, not only leads to the shift of satellite peaks over the range of the coincidence time difference spectrum but also causes a systematic deterioration in energy resolution for shorter delay periods (relative deterioration of about 10%). Additionally, the lowest CRTs (FWHM) achieved vary by up to approximately 30 ps (up to approximately 11% relative change). The lowest FWTM of the coincidence time difference histograms varies by up to approximately 50 ps. For higher overvoltages, the effect of a narrower peaks and less distinct tails in the time difference spectra depending on the delay configurations reverses itself (see Fig. 9b). The computed Gaussian excess factors show that especially for higher overvoltage the influence of non-Gaussian histogram tails become more and more evident (see Fig. 9c). Repeating the measurements and evaluation with different discriminator thresholds *vth*_t1 or narrower coincidence windows did not lead to a change of these effects.

**Fig. 10:**
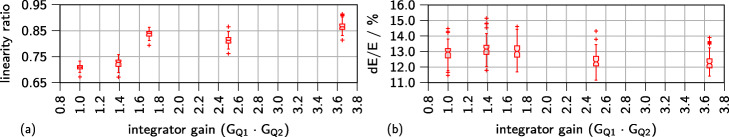
Energy linearity and energy resolution for different integrator gain configurations. The default configuration is *G*_*Q*1_·*G*_*Q*2_=1.0. **a** Energy linearity computed as the ratio of the 511 keV and 1274.5 keV peak positions in the energy value histogram. **b** Energy resolution (dE/E) computed using the FWHM of the 511 keV peak in the energy histogram

Figure 10 depicts the linearity ratio computed as the ratio of the 511 keV and 1275 keV peak positions in the ADC value spectra as well as the energy resolution for different integrator gains *G*_*Q*1_ and *G*_*Q*2_. We find that the default integrator gain setting *G*_*Q*1_=1*.*0 and *G*_*Q*2_=1*.*0 results in the most linear energy spectra (linearity ratio of 0.71, dE/E ≈ 13%). The energy resolution can be improved by approximately 5% (relative improvement) changing the *G*_*Q*1_-setting to *G*_*Q*1_=2*.*5 at the cost of energy linearity. The absolute spread in energy resolution for both of these settings is on the order of approximately 0.6%. The spread is generally increased by higher gains, which also result in a deterioration of the energy resolution to up to 14%.

## Discussion

The peak positions reported for the energy value spectra (see Figs. 4 and 5) suggest that all channels on one ASIC operate uniformly. Since especially in Fig. 4 128 channel, i.e., two ASICs, are considered when computing the channel spread, the operation of two different ASICs is uniform as well. Since single channels not matching the peak position pattern were only identified for one of the Hamamatsu S14161-3050-S-08 SiPM arrays (outliers in Figs. 4a, b), but not for other SiPM arrays, this behavior is probably due to the connected SiPMs and not the operation of ASIC channels. This channel was excluded from further evaluation steps. Since all channels operate uniformly, reporting the CRT and energy resolution as globally computed parameters is justified.

In single- and multi-channel studies, a similar behavior of the CRT and energy resolution dependent on the applied overvoltage was observed. With the selected thresholds, a convergence of the CRT (FWHM) can already be observed for SensL and Hamamatsu SiPMs when setting vth_t1=40 and vth_t1=50 (see Table 2). The improvement observed for KETEK SiPMs is less than 2 ps. For Broadcom SiPMs, a slight convergence is visible yet, as the improvement becomes less for higher thresholds (see Table 5). Additionally, one can observe a slight decrease in energy resolution for higher thresholds (see Tables 4 and 5). This can be explained by the fact that only higher pulses, i.e., events with a higher energy, are triggered on with higher thresholds, leading to more compressed energy spectra. Since for most cases, a convergence of the CRT and a deterioration of the energy resolution is observed, we refrained from triggering on higher thresholds. The non-Gaussian tails of the peak in the coincidence time difference histogram remain below extension 10% of the Gaussian peak at the optimal operation point of the respective SiPM and are suspected to be caused by the detection of scattered events. This matches the observation that the tails are more prone for low thresholds *vth*_t1 and higher overvoltages, increasing the gain of the SiPM, making it more sensitive to noise and low-energy events. Thus, we suspect that the tails could be reduced by applying a narrower energy window. This effect of the energy window on the CRT (FWTM) was investigated for digital SiPMs in [24]. The impact of the discriminator threshold *vth*_t1 on the achieved performance is stronger in multi-channel than in previous single-channel investigations [52]. While in single-channel measurements with two KETEK PM3325-WB-A0 the effect was only visible for overvoltages lower than 3 V and no reverse effect was observed for higher overvoltages, for multi-channel measurements with two KETEK PA3325-WB-0808, the impact of *vth*_t1 was visible over the whole investigated overvoltage range and reversed itself for overvoltages higher than 4 V. The observed differences in behavior could be due to effects on the SiPM array such as crosstalk and light-sharing.

Among the different parameters changed, the scintillator segmentation layer has the largest relative impact on the achieved performance (29% to 43%). The effect of improved performance for BaSO_4_ as reflector material is likely due to a lower probability of light-sharing between channels and thus, a higher fraction of *γ*-events depositing their whole energy on one SiPM channel. This leads to a higher number of small pulses being acquired resulting in a higher mean raw hit rate and especially mean coincidence rate per channel. The observed higher raw data rate with increasing overvoltage is expected due to the increase of the SiPM gain, crosstalk and photo-detection efficiency with the overvoltage (see Fig. 7a). The coincidence rate is expected and observed to be stable over the investigated overvoltage range (see Fig. 7b). Since no saturation is observed, the performance dependency on the reflector material is probably not a *γ*-rate effect. In addition, higher energy values are observed for individual channels if thicker segmentation layers are used as shown in Fig. 4c and d. The different reflective behaviors of BaSO_4_ (diffusive reflection) and ESR foil (specular reflexion) also suspected to contribute to the reduced and increased event rate and more or less light-sharing between single scintillator needles. Setting higher thresholds *vth*_t2 and *vth*_e could be used to filter out these events, while still triggering on the first optical photons with a low thresholds *vth*_t1, which preserve the ToF information of the detected event. An improved performance seen in experiments with larger distances to the employed sources, which also is associated with a lower mean raw hit rate per channel and lower mean coincidence rate per channel (see Fig. 7c, d), additionally indicates a raw-data-rate-dependent performance. Here, a saturation of the mean raw hit rate per channel is visible for the detector position closest to the sources at higher overvoltage (see Fig. 7c). The detected coincidence rate drops accordingly (see Fig. 7d). Since here, the detector configuration stayed the same and only the distance to the source was varied, this is a *γ*-rate effect. Since the origin of the loss of events is not flagged, it cannot be determined if this drop is due to the ASIC or other parts of the signal chain. PETsys Electronics S.A. states a maximum event rate of up to 480 kcps per channel [49], which is not reached by the detected mean raw hit rate per channel.

Communication with PETsys lead to the assumption that the systematic deterioration in energy resolution with changing the trigger delay period is probably related to the configuration of the energy integration window. Acquiring data in qdc-mode, the integrated charge is corrected by an estimate for a charge offset related to the time difference between *trigger*_Q, which starts the energy integration, and the delayed *trigger*_T1, which sets the event timestamp, i.e., the time of arrival of the optical photons hitting the SiPM channel. The latter one is prolonged or shortened with respect to the configuration of the trigger delay period, which results in an adaptation of the charge offset applied as a correction. The energy integration window is not adapted for different trigger delay periods and thus, needs to be adjusted manually. In all investigations so far, this window has been kept at a fixed default value of approximately 290 ns. Further experiments to investigate the influence of manually adjusting the window are on-going. The difference in behavior as a function of overvoltage cannot be understood without profound knowledge of the details of the ASIC implementation. Discussions with PETsys regarding this matter are on-going.

Investigations with two Hamamatsu S14161-3050-HS-08 arrays show that the integrator gain setting can be used to linearize the acquired energy spectra and optimize the energy resolution. The absolute spread in the energy resolution of all investigated channels is in the order of less than 1%. Thus, it can be concluded that the SiPM and ASIC channels operated uniformly in this experiment. In this study, the ASIC is already operated at the most linear setting possible. This setting is recommended to be used for further investigations featuring high-gain SiPMs. Switching to a more non-linear setting to improve the energy resolution at 511 keV is possible as long as the saturation correction method can be applied, i.e., as long as the two peaks of the Na^22^-spectrum remain separable.

Comparing the performance of different analog SiPM types, Hamamatsu S14161-3050-HS-08 and Broadcom AFBR-S4N44P643S show the lowest CRTs in combination with the TOFPET2 ASIC. Both SiPM types feature a higher gain and a larger SPAD size than the investigated KETEK and SensL SiPMs (see Table 1). However, it has to be kept in mind that this study only reports performance results for the combination of SiPM type and ASIC using the delivered benchtop setup “as-is.” Since the coupling between SiPM and ASIC was not individually optimize for each SiPM type, this study cannot be used to compare the performance of solely the SiPM types among each other, but only their performance in combination with the TOFPET2 ASIC for the specific coupling scheme given by the electronics provided. Individual optimization of the SiPM-ASIC coupling is expected to improve the reported performance [69, 70].

On benchtop level, the TOFPET2 ASIC measures up to the performance of state-of-the-art ASICs in single-channel coincidence experiments. An overview on single-channel experiments is provided in [52]. In comparison with other multi-channel performance experiments, the TOFPET2 ASIC measures up to state-of-the-art system performance regarding its ToF capability. An 18-channel ASIC using a tot-method to digitize energies achieved a CRT of 275 ps and an energy resolution of 11.8% in coincidence experiments with two 12 × 12 Hamamatsu C13500-4075LC-12 modules coupled to 20-mm-high scintillator arrays and read out by 8 ASICs in total [71]. Module tests with the PETA5 ASIC and SiPMs fabricated by Fondazione Bruno Kessler (FBK, RGB-HD technology) achieved and average CRT of 230 ps and an energy resolution of about 14% when one-to-one-coupled to 10-mm-high LYSO scintillator arrays [47]. PETsys reports a CRT of 260 ps between two Hamamatsu S13361-3050AE-04 arrays each one-to-one-coupled to an array of 15-mm-high LYSO needles [12]. On system level, the TRIMAGE scanner equipped with the TRIROC ASIC reaches CRTs of 515 ps and an energy resolution between 20% to 22% employing a two-layered scintillator geometry consisting of LYSO needles with a total height of 20 mm on FBK SiPMs (NUV-HD technology) [72]. A study on a prototype PET scanner employing digital SiPMs (DPC 3200-22 by Philips Digital Photon Counting) coupled to 10-mm-high LYSO scintillator arrays reports CRTs down to 215.2 ± 0.4 ps (energy resolution: 11.381 ± 0.007%) [11]. The Biograph Vision PET/CT (computed tomography) scanner recently released by Siemens also achieves 214 ps on system level, using 3.2 mm × 3.2 mm × 20 mm LSO needles [73, 74]. Further performance studies should include monolithic scintillator blocks as well as slabs to obtain DOI information with the TOFPET2 ASIC. First experiments featuring DOI-capable scintillator geometries have been conducted [22, 55, 56].

In addition, when designing a PET system or a MR-compatible PET insert, one has to keep in mind other system requirements such as power supply limitations or interference problems [75]. The power consumption of the TOFPET2 ASIC and its influence on the ASIC performance were already evaluated in an additional study [59]. Regarding MR-compatibility tests, similar test protocols as in [28, 76, 77] need to be defined and executed.

## Outlook and conclusion

Generally, the TOFPET2 ASIC evaluation kit enables the user to easily test various SiPM types and scintillator topologies in combination with the TOFPET2 ASIC.

The ASIC is compatible with all SiPM types tested in this study (see Table 1). We observe that high discriminator thresholds (*vth*_t1=40−50), thicker reflector layers, and lower data rates (larger distances to the sources and diffusive inter-crystal reflexion) result in an improved performance regarding both CRT and energy resolution. Among the SiPM types used, so far, Broadcom AFBR-S4N44P643S and Hamamatsu S14161-3050-HS-08 SiPMs show the most promising performance results in combination with the TOFEPT2 ASIC.

The appearance of satellite peaks is confirmed for multi-channel time difference spectra. A performance dependency related to the configuration of the delay element is observed. A first indication of a *γ*-rate-dependent ASIC performance was found in experiments with different distances to a Na^22^ source. Adjustments of the integrator gain can be used to linearize the energy value spectra acquired in qdc-mode or to improve the energy resolution.

Even without optimizing the SiPM-to-ASIC coupling, the CRTs and energy resolutions reported for a coincidence setup of two detector blocks lay in the order of the performance of clinical and pre-clinical ToF-PET systems, while providing a uniform and stable readout of multiple channels at the same time. Therefore, it will be considered for integration in (whole-body) ToF-PET/MRI applications.

## Data Availability

The datasets used and/or analyzed during the current study are available from the corresponding author on reasonable request.

## Abbreviations

ASIC: application-specific integrated circuit
BGO: bismuth germanate
CRT: coincidence resolution time
CT: computed tomography DOI: depth of interaction
FBK: Fondazione Bruno Kessler
FEB: front end board
FWHM: full width at half maximum
FWTM: full width at tenth maximum
GbE: Gigabit Ethernet
HV-DAC: high-voltage digital-to-analog converter
LOR: line of response
LYSO: lutetium-yttrium oxyorthosilicate
MRI: magnetic resonance imaging
PDE: photo detection efficiency PET: positron emission tomography
QDC: charge-to-digital converter
SiPM: silicon-photomultiplier
TDC: time-to-digital converter
ToF: time-of-flight
